# Stand‐alone model for delivery of oral HIV pre‐exposure prophylaxis in Kenya: a single‐arm, prospective pilot evaluation

**DOI:** 10.1002/jia2.26131

**Published:** 2023-06-12

**Authors:** Katrina F. Ortblad, Peter Mogere, Victor Omollo, Alexandra P. Kuo, Magdaline Asewe, Stephen Gakuo, Stephanie Roche, Mary Mugambi, Melissa Latigo Mugambi, Andy Stergachis, Josephine Odoyo, Elizabeth A. Bukusi, Kenneth Ngure, Jared M. Baeten

**Affiliations:** ^1^ Public Health Sciences Division Fred Hutchinson Cancer Center Seattle Washington USA; ^2^ Centre for Clinical Research Kenya Medical Research Insititute Nairobi Kenya; ^3^ Centre for Microbiology Research Kenya Medical Research Insititute Nairobi Kenya; ^4^ Department of Pharmacy University of Washington Seattle Washington USA; ^5^ National AIDS and STI Control Programme Kenya Ministry of Health Nairobi Kenya; ^6^ Department of Global Health University of Washington Seattle Washington USA; ^7^ Department of Obstetrics and Gynecology University of Washington Seattle Washington USA; ^8^ School of Public Health Jomo Kenyatta University of Agriculture and Technology Nairobi Kenya; ^9^ Department of Epidemiology University of Washington Seattle Washington USA; ^10^ Department of Medicine University of Washington Seattle Washington USA

**Keywords:** differentiated service delivery (DSD), HIV prevention, Kenya, pre‐exposure prophylaxis (PrEP), private pharmacies, retention

## Abstract

**Introduction:**

The delivery of daily, oral HIV pre‐exposure prophylaxis (PrEP) at private pharmacies may overcome barriers to PrEP delivery at public healthcare facilities, including HIV‐associated stigma, long wait times and overcrowding.

**Methods:**

At five private, community‐based pharmacies in Kenya, a care pathway for PrEP delivery (ClinicalTrials.gov: NCT04558554) was piloted—the first of its kind in Africa. Pharmacy providers screened clients interested in PrEP for HIV risk, then used a prescribing checklist to identify clients without medical conditions that might contraindicate PrEP safety, counsel them on PrEP use and safety, conduct provider‐assisted HIV self‐testing and dispense PrEP. For complex clinical cases, a remote clinician was available for consultation. Clients who did not meet the checklist criteria were referred to public facilities for free services delivered by clinicians. Pharmacy providers dispensed a 1‐month PrEP supply at initiation and a 3‐month supply thereafter at a client fee of 300 KES (∼$3 USD) per visit.

**Results:**

From November 2020 to October 2021, pharmacy providers screened 575 clients, identified 476 who met the prescribing checklist criteria and initiated 287 (60%) on PrEP. Among pharmacy PrEP clients, the median age was 26 years (IQR 22–33) and 57% (163/287) were male. The prevalence of behaviours associated with HIV risk among clients was high; 84% (240/287) reported sexual partners with unknown HIV status and 53% (151/287) reported multiple sexual partners (past 6 months). PrEP continuation among clients was 53% (153/287) at 1 month, 36% (103/287) at 4 months and 21% (51/242) at 7 months. During the pilot observation period, 21% (61/287) of clients stopped and restarted PrEP and overall pill coverage was 40% (IQR 10%–70%). Nearly, all pharmacy PrEP clients (≥96%) agreed or strongly agreed with statements regarding the acceptability and appropriateness of pharmacy‐delivered PrEP services.

**Conclusions:**

Findings from this pilot suggest that populations at HIV risk frequently visit private pharmacies and PrEP initiation and continuation at pharmacies is similar to or exceeds that at public healthcare facilities. Private pharmacy‐based PrEP delivery, conducted entirely by private‐sector pharmacy staff, is a promising new delivery model that has the potential to expand PrEP reach in Kenya and similar settings.

## 1 INTRODUCTION

Despite the availability of free HIV testing, treatment and daily, oral pre‐exposure prophylaxis (PrEP) at public healthcare facilities in many sub‐Saharan African countries [1, 2], HIV incidence persists above levels of epidemic control [3]. Both client‐ and provider‐level barriers to facility‐delivered PrEP services limit the reach of this highly effective prevention intervention [4, 5, 6, 7]. Client‐level barriers include the stigma associated with visiting facility‐based HIV clinics [8], limited hours of facility operation [9] and long travel distances to and time waiting at healthcare facilities [8], while provider‐level barriers include limited time, overcrowding [10], organizational prioritization of curative over preventive care [11, 12] and inadequate PrEP knowledge [13]. Innovative PrEP delivery models are needed to address access and delivery barriers and expand PrEP services to populations who could benefit [14].

Private, community‐based pharmacies, which are ubiquitous in sub‐Saharan Africa and often a first point of healthcare access [15, 16, 17], are well‐suited for addressing some barriers to facility‐delivered PrEP services. Pharmacies already provide many preventive and curative sexual and reproductive health services (e.g. contraception and sexually transmitted infection [STI] treatment), with pharmacy customers frequently opting to purchase services at pharmacies that are available for free at public facilities (e.g. pregnancy testing) [15]. In preparatory formative research, pharmacy providers and clients in Kenya anticipated that pharmacy‐delivered PrEP services would have advantages over facility‐delivered services in the short‐term (e.g. increased convenience and privacy) and long‐term (e.g. decreased facility congestion) and that they would accept this model so long as the services delivered were high‐quality [8].

Since beginning national PrEP rollout in 2017 [18], Kenya has been a leader in developing and testing differentiated models of PrEP service delivery, principally through public‐sector HIV and primary healthcare clinics [19]. To reach populations that do not commonly seek health services in the public sector, Kenya's National AIDS & STI Control Program (NASCOP) called for evidence on the effectiveness and safety of PrEP delivery at private pharmacies. In collaboration with Kenyan stakeholders, a model of pharmacy‐delivered PrEP services was developed to implement for research purposes [10, 20]—one of the first of its kind in Africa [21]. This model was adapted from a model ongoing in the United States (Seattle, Washington) [22]; details of the design process are published elsewhere [23].

## 2 METHODS

### 2.1 Pilot pharmacies and providers

A single‐arm, prospective pilot evaluation of pharmacy‐delivered PrEP services was conducted at five private pharmacies in Kenya (ClinicalTrials.gov NCT04558554): three in Kiambu County (4% HIV prevalence) and two in Kisumu County (16% HIV prevalence) [24]. Eligible pharmacies were registered with the Kenya Pharmacy and Poisons Board, had a full‐time licensed pharmacist or pharmaceutical technologist, had a private room for counselling and HIV testing, and were willing to participate in research activities. Prior to implementation, the lead provider at each pharmacy completed a 2‐day training on the core components of pharmacy‐delivered PrEP services. The training was virtual (via Zoom) because implementation began in October 2021 during the COVID‐19 pandemic. Following the training, providers received weekly on‐site technical assistance by research staff, which was phased out over time.

Three months into pilot implementation, one pharmacy owner in Kiambu County stopped participation due to concerns about their business being associated with HIV service delivery. After this pharmacy was replaced, it did not initiate new clients on PrEP but continued serving existing ones.

### 2.2 Participants

To recruit participants, promotional posters encouraging clients to ask about PrEP for HIV prevention were placed at the pharmacies (Figure S1). Additionally, pharmacy providers were trained to offer PrEP eligibility screening to clients seeking commodities indicative of sexual activity and potential HIV exposure (e.g. emergency contraception, pregnancy testing and STI treatment). Eligible clients were ≥18 years old, met the criteria on a standardized PrEP prescribing checklist and were willing to participate in research activities. Each pilot pharmacy was encouraged to initiate up to 75 PrEP clients, after which PrEP‐interested clients were referred to public healthcare facilities.

### 2.3 Procedures

**Figure 1:**
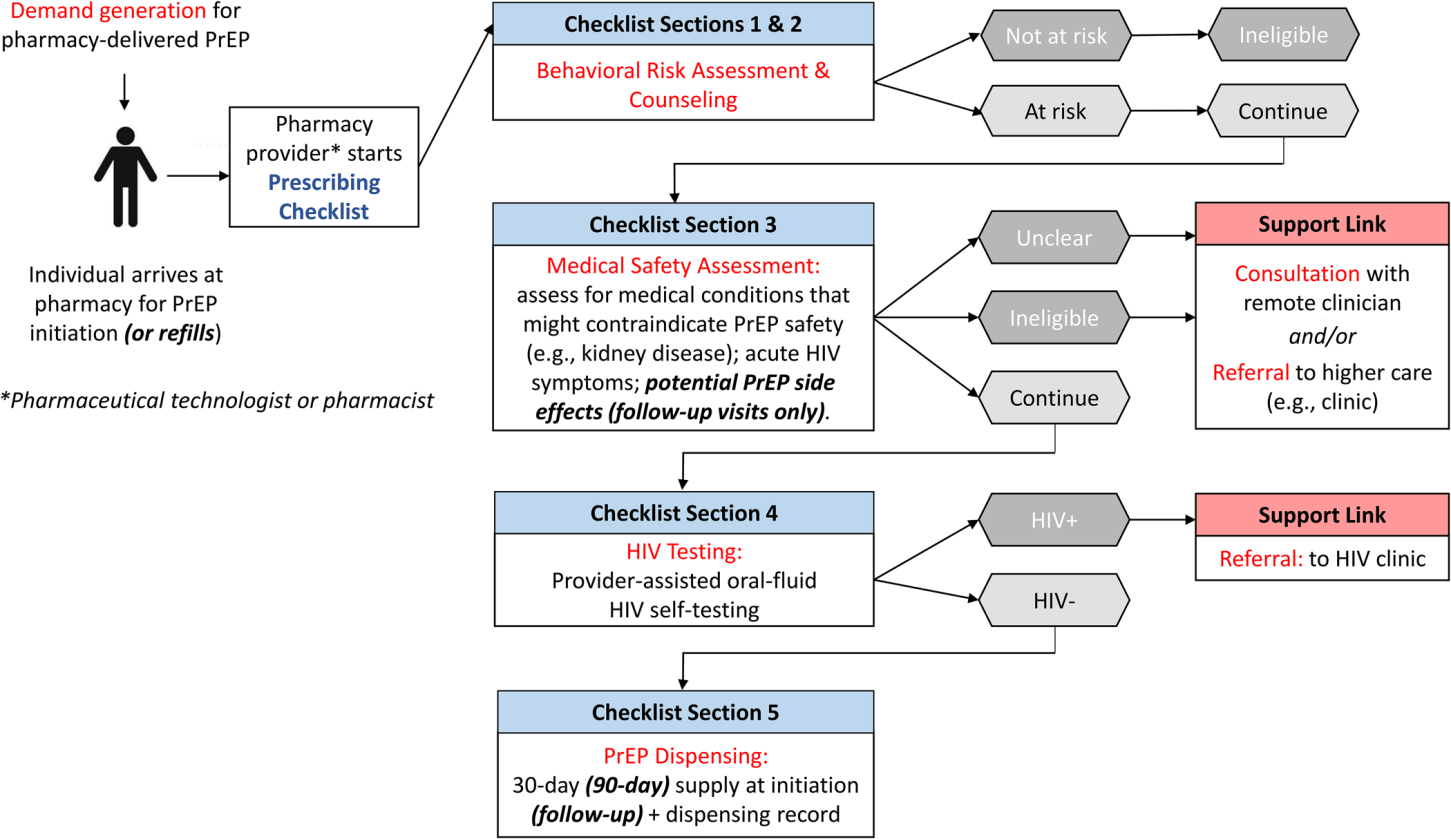
Care pathway for pharmacy‐delivered oral PrEP services. Abbreviation: PrEP, pre‐exposure prophylaxis.

Participating pilot pharmacies delivered PrEP services according to Kenya's national PrEP delivery guidelines [25]. For the purposes of this research, Kenyan regulatory agencies permitted trained pharmacy providers to use a pre‐approved prescribing checklist (Figure S2) to determine PrEP eligibility and dispense PrEP without a prescription from a doctor or nurse. At any time, providers could consult a remote clinician (employed by the Kenyan research team) for questions or client referrals via phone or a private WhatsApp group. The prescribing checklist guided pharmacy providers through the following core components of the delivery model (Figure 1).

### 2.4 Behavioural risk assessment and associated counselling services

Pharmacy providers used a modified version of Kenya's PrEP Rapid Assessment Screening Tool (RAST), an eight‐item (modified to 10‐item) questionnaire routinely used at public healthcare facilities to determine HIV risk and PrEP eligibility [18]. The RAST includes questions about clients’ self‐reported HIV status and that of their sexual partner(s) as well as behaviours in the past 6 months associated with the risk of HIV acquisition (e.g. condomless sex with a partner of unknown HIV status). Providers used clients’ RAST responses to tailor counselling on the potential benefits of PrEP for HIV prevention, importance of adherence and potential side effects.

### 2.5 Medical safety assessment

Pharmacy providers screened clients for a history of liver disease, kidney disease or diabetes—all medical conditions that might contraindicate PrEP safety [26]. Clients who reported any of these conditions were determined too clinically complex for pharmacy‐delivered PrEP services and referred to a nearby public healthcare facility for further evaluation by a clinician. Pregnant or breastfeeding women were additionally referred to facility‐delivered PrEP services, as the management of PrEP and pregnancy was beyond the scope of practice for pharmacy providers in this pilot. Pharmacy providers also screened for acute symptoms of HIV infection at all visits and for potential PrEP side effects at follow‐up visits.

### 2.6 HIV testing

After confirming PrEP eligibility and safety, pharmacy providers assisted clients with oral‐fluid HIV self‐testing (HIVST) (OraQuick In‐Home HIV Test, OraSure Technologies, USA) in a private room to confirm HIV‐negative status. Although not in the national guidelines [27, 28], NASCOP permitted the use of provider‐assisted HIVST for PrEP initiation and continuation in this pilot, as it was the only form of HIV testing allowed at private pharmacies at the time [10]. Clients who tested HIV positive were referred to public healthcare facilities for confirmatory testing and treatment, while those who tested HIV negative were dispensed PrEP. Clients were not tested for hepatitis B or C and did not have their kidney function (i.e. creatinine clearance) tested, as these tests are rarely available at public healthcare facilities in Kenya and the national PrEP delivery guidelines state that their lack of availability should not delay PrEP initiation [25].

### 2.7 PrEP dispensing

At initiation visits, clients received a 1‐month supply of daily, oral PrEP and were scheduled for follow‐up 1 month later. Pharmacy providers did not remind clients due for PrEP refills but called or texted clients if they missed their follow‐up visit, as they typically do for clients using chronic medications. At all follow‐up visits, clients who continued to meet the criteria on the prescribing checklist, including testing HIV negative, received a 3‐month PrEP supply and were scheduled for follow‐up 3 months later. All clients were eligible for up to three PrEP follow‐up visits, then were referred to public healthcare facilities for continued PrEP services.

Pilot pharmacies secured PrEP medication and HIVSTs from Kenya Ministry of Health (MOH) stores at no cost. At each visit, pharmacies charged clients a fee of 300 Kenyan shillings (KES) (∼$3 US dollars [USD]) for providers’ time delivering of PrEP services. This fee was determined via consensus with participating pharmacy providers. Additionally, each pilot pharmacy was compensated 5000 KES (∼$50 USD) monthly for engagement in research activities (e.g. allowing a research assistant to be stationed at the pharmacy to complete research‐related activities).

### 2.8 Data collection

Research assistants consented eligible clients and, at the end of each visit, administered questionnaires. The questionnaires collected information on clients’ socio‐demographic characteristics, healthcare‐seeking behaviours, sexual behaviours, self‐reported PrEP adherence (follow‐up visits only), and experiences and perceptions of pharmacy‐delivered PrEP services. Research assistants did not participate in PrEP delivery.

### 2.9 Utilization outcomes

The primary pilot study outcomes were PrEP initiation, continuation and adherence. PrEP initiation was measured among all clients who met the prescribing checklist criteria; those dispensed PrEP were categorized as having initiated. PrEP continuation and adherence were measured at scheduled follow‐up visits (i.e. 1, 4 and 7 months) among clients who initiated PrEP [29, 30, 31, 32]. To continue PrEP, clients had to refill PrEP (i.e. met the prescribing checklist criteria and be dispensed PrEP) at least 15 days prior to their next scheduled visit. The PrEP continuation measurement was then divided into two categories: (1) on‐time continuation (refilling PrEP within 15 days after a scheduled visit), and (2) stopping and restarting PrEP (refilling PrEP more than 15 days after a scheduled visit) [33]. During implementation, all clients were counselled on consistent PrEP use.

PrEP adherence was assessed via self‐reported measures and pill coverage. At each pharmacy PrEP visit, clients were asked to report the number of pills missed, ability to use PrEP and frequency of PrEP use in the past month [34]. The ability to use and frequency of use questions were assessed using 5‐point Likert scales and responses were transformed to 100‐point scales, with higher scores indicating better adherence [35]. Pill coverage was measured by dividing the total number of PrEP pills dispensed per client by their number of days of potential HIV risk exposure (maximum of 300 days/client), with the assumption that clients who initiated PrEP remained at HIV risk throughout the pilot duration.

### 2.10 Implementation outcomes

At each study visit, client perceptions of the acceptability and appropriateness of pharmacy‐delivered PrEP services were assessed. Acceptability—the perception an intervention is agreeable, palatable or satisfactory [36]—was measured using four statements that assess different component constructs of the Theoretical Framework of Acceptability (e.g. affective attitude and burden) [37]. Appropriateness—the perceived fit, relevance or compatibility of an intervention [36]—was measured using two statements modified from the Intervention Appropriateness Measure [38]. For each statement, clients indicated their level of agreement on a 5‐point Likert scale; if >80% of clients “agreed” or “strongly agreed” with a statement, we categorized that construct as achieved [39]. Additionally, at each follow‐up visit, clients were asked how much they would be willing to pay for a pharmacy PrEP visit that included counselling, a medical safety assessment, HIV testing and a 3‐month PrEP supply.

### 2.11 Analyses

Outcomes were reported descriptively for all clients and four subgroups: women <25, men <25, women ≥25 and men ≥25 years old. Chi‐squared tests were conducted to determine if differences in proportions between the subgroups were significant (*p*<0.05). Poisson regression models, clustered by pharmacy, were used to identify correlates of any PrEP continuation and any stopping and restarting PrEP. In these analyses, prevalence ratios were calculated with 95% confidence intervals and a defined significance threshold of *p*<0.05. All multivariable analyses included age, sex, marital status and having a partner of unknown HIV status, as well as select demographic and behavioural characteristics (e.g. RAST items with a baseline prevalence >10%) associated with the outcomes at a threshold of *p*<0.10 in univariable analyses. StataSE 16 was used for all analyses.

### 2.12 Ethics

The study protocol was reviewed and approved by the Scientific Ethics Review Unit at the Kenya Medical Research Institute and the Human Subjects Division at the University of Washington. All participants provided written informed consent and received 500 KES (∼$5 USD) for the completion of research activities (e.g. questionnaires).

## 3 RESULTS

### 3.1 PrEP initiation

**Figure 2:**
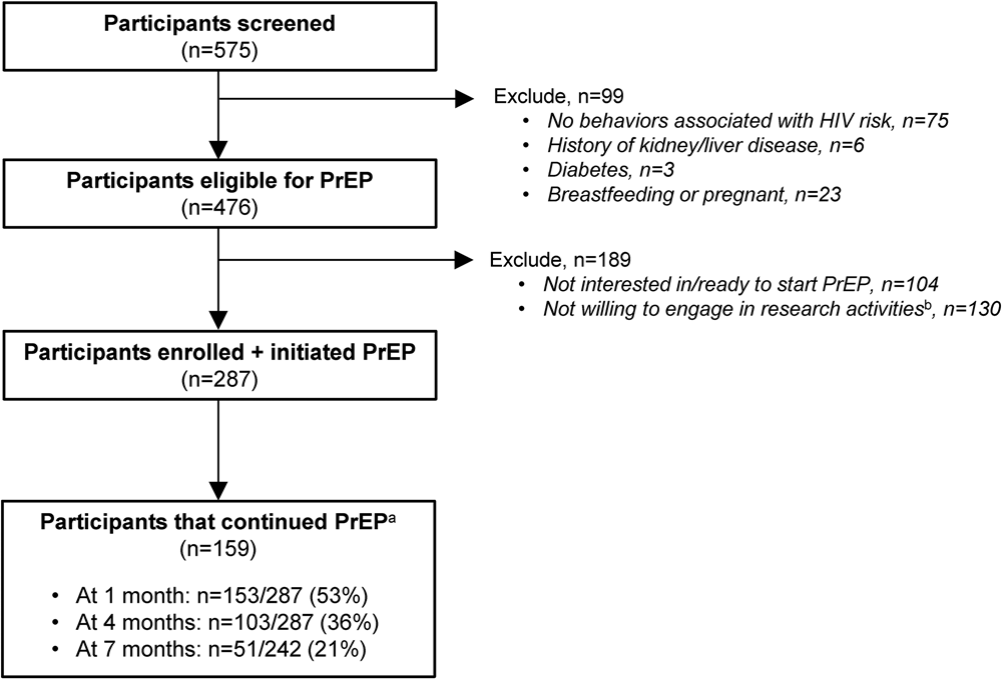
CONSORT diagram. ^a^Percentages are calculated among those eligible for each visit. ^b^This includes both “Not willing to consent in pilot activities,” *n* = 123 and “Not willing to be counselled on PrEP delivery,” *n* = 106.

From November 2020 to December 2021, 575 pharmacy clients were offered PrEP screening, 476 were determined PrEP‐eligible and 287 (60% of those eligible) initiated PrEP (Figure 2). Among the 99 clients determined ineligible, 75 (76%) did not have any behaviours associated with HIV risk, nine (9%) reported medical conditions that might contraindicate PrEP safety and 23 (23%) were pregnant or breastfeeding. No clients tested positive for HIV during PrEP eligibility screening.

Among clients who initiated PrEP, over half were men (57%, 163/287), almost half were <25 years old (44%, 126/287) and less than half were married (38%, 108/287) (Table 1). Most PrEP clients (58%, 166/287) travelled <15 minutes to the pharmacy, and many (54%, 155/287) reported that private pharmacies are typically the first place they seek healthcare. Only two clients (1%) reported any prior PrEP use. Most clients learned of pharmacy‐delivered PrEP services by informal word‐of‐mouth referral (43%, 123/287) or the pharmacy provider (42%, 121/287).

**Table 1:** Characteristics of pharmacy clients initiating PrEP, *N*=287

Characteristic	*N* (%)
Pharmacy	
*Kisumu: Pharmacy A*	73 (25%)
*Kisumu: Pharmacy B*	73 (25%)
*Thika: Pharmacy C*	57 (20%)
*Thika: Pharmacy D*	29 (10%)
*Thika: Pharmacy E*	55 (19%)
Demographics	
Age, median (IQR)	26 (22−33)
* <25 years*	126 (44%)
Sex	
* Female*	124 (43%)
* Male*	163 (57%)
Years of schools, median (IQR)	14 (12−16)
Currently in school	79 (28%)
Married	108 (38%)
Relationship status	
* Single*	2 (1%)
* Casual partner(s) only*	96 (33%)
* Primary partner only*	88 (31%)
*Primary & casual partners*	101 (35%)
Monthly individual income, median KES (IQR)^a^	6000 (0−15,000)
Pharmacy access	
Travel time to pharmacy	
* <5 minutes*	27 (9%)
* 5 to <15 minutes*	139 (48%)
* 15 to <30 minutes*	81 (28%)
* ≥30 minutes*	40 (14%)
Private pharmacy is first place of healthcare seeking	155 (54%)
Pharmacy visits per month, median (IQR)	1 (0−1)
Sexual and reproductive health	
Was seeking an SRH service^b^	261 (91%)
Uses LARC or hormonal form of contraception^b^, ^c^	45 (16%)
Has used emergency contraception ≥2 times^d^	57 (46%)
Trying to conceive	29 (10%)
Has tested for HIV	268 (93%)
* Months since last test, median (IQR)*	6 (3−12)
PrEP knowledge	
Knows someone using PrEP	106 (37%)
* Prior PrEP use*	2 (1%)
How learned of pharmacy PrEP	
* Pharmacy provider*	121 (42%)
* Informal word‐of‐month*	123 (43%)
* Poster*	26 (9%)
* Other*	33 (11%)

Abbreviations: IQR, interquartile range; KES, Kenyan shilling; LARC, long‐acting forms of contraception; SRH, sexual and reproductive health service.

^a^ USD equivalent is $56.40 ($0−$141). Converted from KES to USD using conversion rate averaged from 11/2020 to 12/2021 ($1USD = 110.72 KES); https://www.exchangerates.org.uk/KES‐USD‐spot‐exchange‐rates‐history‐2020.html.

^b^ SRH services sought included PrEP (63%, *n*=181), HIV self‐testing (20%, *n*=57), family planning (13%, *n*=38), pregnancy testing (3%, *n*=10) or sexual performance enhancing drug (3%, *n*=9).

^c^ Included the following forms of contraception: implant (7%, *n*=21), injectable (5%, *n*=13), oral pill (3%, *n*=8) and intrauterine device (IUD) (1%, *n*=3).

^d^ Reported among female participants only (*n*=124).

**Figure 3:**
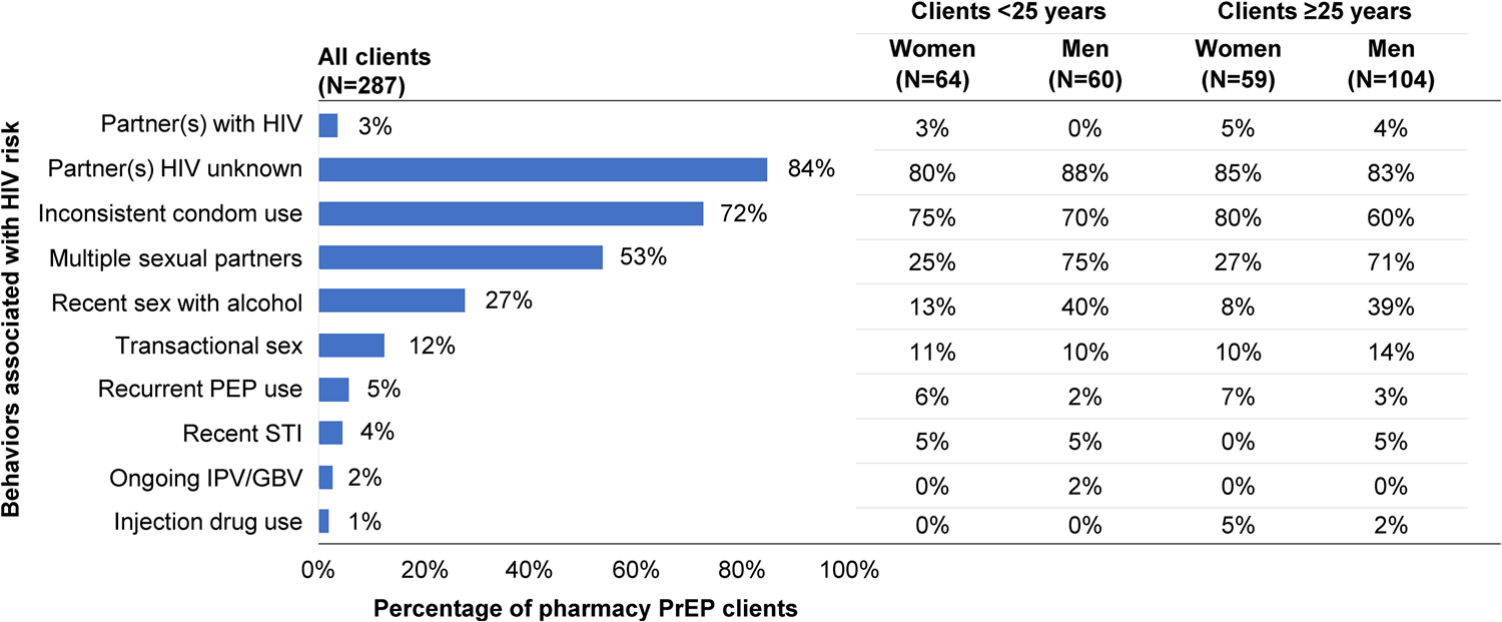
Behaviours associated with HIV risk acquisition among pharmacy clients initiating PrEP. Abbreviations: GBV, gender‐based violence; IPV, intimate partner violence; PEP, post‐exposure prophylaxis; PrEP, pre‐exposure prophylaxis; STI, sexually transmitted infection.

The prevalence of behaviours associated with HIV risk acquisition among clients who initiated PrEP was high, especially among male clients (Figure 3). Among all PrEP clients, most (84%, 241/287) reported partners with unknown HIV status, many (72%, 207/287) reported inconsistent condom use and half (53%, 152/287) reported multiple sexual partners in the past 6 months. Compared to female clients, male clients reported a significantly higher prevalence of multiple sexual partners (*p*<0.001) and recent engagement in sex with alcohol (*p*<0.001).

### 3.2 PrEP continuation and adherence

**Figure 4:**
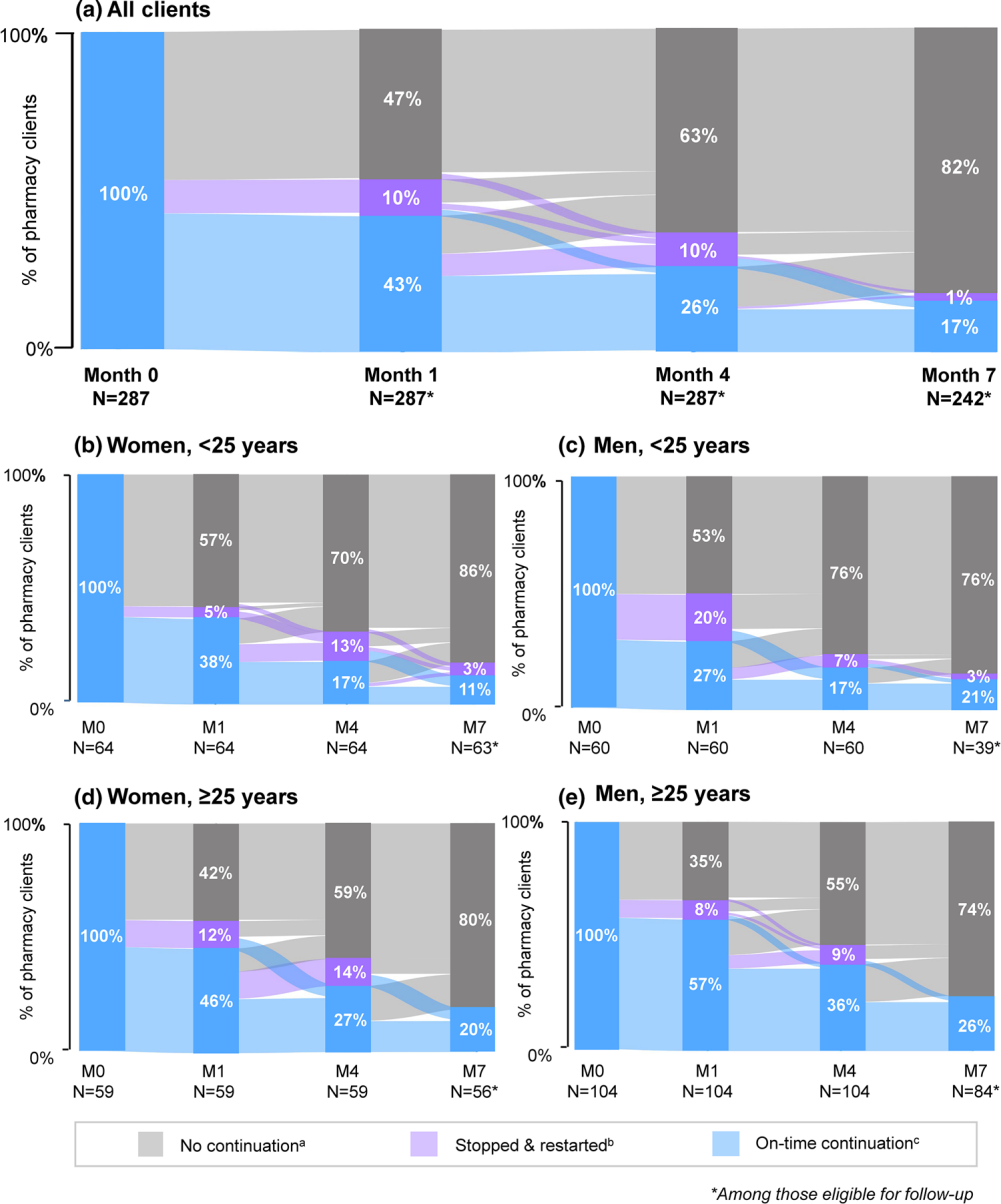
PrEP continuation at 1, 4 and 7 months following initiation among pharmacy clients. Percentages are calculated among those eligible for each visit. Abbreviation: M, month. ^a^No continuation: pharmacy clients did not return to any follow‐up visit nor refilled their PrEP medication. ^b^Stopped & restarted: pharmacy clients returned to their follow‐up visit outside of their retention window (i.e. over 15 days after/prior to the next scheduled follow‐up visit) and refilled their PrEP medication. ^c^On‐time continuation: pharmacy clients both returned to the follow‐up visit and refilled their PrEP medication within the study retention window (i.e. up until 15 days until the next scheduled visit).

PrEP continuation among clients who initiated PrEP was 53% (153/287) at 1 month, 36% (103/287) at 4 months and 21% (51/242) at 7 months (Figure 4). PrEP continuation varied among the subgroups, with significantly higher continuation among those ≥25 years compared to those <25 years (*p*<0.001). At 1 month, men ≥25 years had the highest PrEP continuation (64%, 67/104) and women <25 years had the lowest continuation (42%, 27/64) (*p*<0.005)—a pattern that remained consistent at 7 months. While most clients who returned for their 1‐month visit did so on time (43%, 66/153), a higher percentage of young women who returned did so on time (89%, 24/27) compared to the other subgroups (*p*<0.001). Self‐reported PrEP adherence was high across all pharmacy clients and subgroups—findings that remained consistent across follow‐up visits (Table 2).

**Table 2:** Self‐reported PrEP adherence at 1, 4 and 7 months among pharmacy clients

	Month 1	Month 4	Month 7
All participants (*N*=287):	*N*=287	*N*=287	*N*=242
Retained in care,^a^ *n* (%)	153 (53%)	103 (36%)	51 (21%)
PrEP adherence score, past month^b^	89 (78−100)	89 (78−100)	91 (80−100)
* # missed pills, median (IQR)*	2 (0−5)	2 (0−5)	1 (0−3)
* Ability to use, median (IQR)* *^c^*	75 (75−100)	75 (75−100)	75 (75−100)
* Frequency of use, median (IQR)* *^d^*	100 (75−100)	100 (75−100)	100 (75−100)
**Clients <25 years**:			
**Women (*N*=64)**	***N*=64**	***N*=64**	***N*=63**
Retained in care,^a^ *n* (%)	27 (42%)	19 (30%)	9 (14%)
PrEP adherence score, past month^b^	89 (78−100)	89 (78−100)	86 (80−100)
* # missed pills, median (IQR)*	2 (0−5)	2 (0−5)	1 (0−3)
* Ability to use, median (IQR)* *^c^*	75 (75−100)	75 (75−100)	75 (75−100)
* Frequency of use, median (IQR)* *^d^*	100 (75−100)	100 (75−100)	87.5 (75−100)
**Men (*N*=60)**	***N*=60**	***N*=60**	***N*=39**
Retained in care,^a^ *n* (%)	27 (45%)	14 (23%)	9 (23%)
PrEP adherence score, past month^b^	80 (73−99)	89 (79−94)	99 (83−100)
* # missed pills, median (IQR)*	3 (1−7)	2 (0−4)	1 (0−2)
* Ability to use, median (IQR)* *^c^*	75 (69−100)	75 (75−81)	100 (69−100)
* Frequency of use, median (IQR)* *^d^*	75 (75−100)	100 (75−100)	100 (88−100)
**Clients ≥25 years**:			
**Women (*N*=59)**	***N*=59**	***N*=59**	***N*=56**
Retained in care,^a^ *n* (%)	34 (58%)	24 (41%)	11 (20%)
PrEP adherence score, past month^b^	91 (79−100)	89 (78−100)	91 (81−100)
* # missed pills, median (IQR)*	1 (0−4)	2 (0−5)	1 (0−2)
* Ability to use, median (IQR)* *^c^*	75 (75−100)	75 (75−100)	75 (75−100)
* Frequency of use, median (IQR)* *^d^*	100 (75−100)	100 (75−100)	100 (75−100)
**Men (*N*=104)**	***N*=104**	***N*=104**	***N*=84**
Retained in care,^a^ *n* (%)	67 (64%)	46 (44%)	22 (26%)
PrEP adherence score, past month^b^	89 (76−100)	98 (80−100)	99 (80−100)
* # missed pills, median (IQR)*	2 (0−7)	2 (0−3)	1 (0−3)
* Ability to use, median (IQR)* *^c^*	75 (75−100)	100 (75−100)	100 (75−100)
* Frequency of use, median (IQR)* *^d^*	100 (75−100)	100 (75−100)	100 (75−100)

Abbreviation: IQR, interquartile range.

^a^ We defined “retained in care” as returning for a PrEP follow‐up visit more than 15 days prior to the next scheduled visit date.

^b^ The PrEP adherence score is a validated 100‐point scale that is an average participants’ responses to three questions on PrEP adherence in the past 30 days: (a) number of missed taken [(30 days−number of missed pills)*3.33], (b) self‐reported PrEP adherence and (c) self‐reported frequency of PrEP use. PrEP adherence score = (a + b + c )/3 (Wilson I. *Current HIV/AIDS Reports* 2009).

^c^ “Please rate your ability in the past month to take the PrEP pills exactly as you were instructed.” Likert scale (0 = very poor, 1 = poor, 2 = fair, 3 = good, 4 = excellent) was converted to 100‐point scale by multiplying values by 25 (e.g. 100 points = excellent). (Wilson I. *Current HIV/AIDS Reports* 2009).

^d^ “In the past month, how often did you take your PrEP pills the way you were supposed to?” Likert scale (0 = never, 1 = rarely, 2 = sometimes, 3 = usually, 4 = always) was converted to 100‐point scale by multiplying values by 25 (e.g. 100 points = excellent). (Wilson I. *Current HIV/AIDS Reports* 2009).

Any PrEP continuation across all follow‐up visits was 53% (153/287) among pharmacy PrEP clients, with 36% (103/287) of clients returning for at least two follow‐ups and 21% (51/242) returning for three follow‐up visits (Table 3). Additionally, 21% (60/287) of clients stopped and restarted PrEP at some point during follow‐up. Older men had the highest overall PrEP continuation (64%, 67/104), and young women had the lowest (45%, 27/60) (*p*<0.001). Younger men had the highest prevalence of stopping and restarting PrEP (57%, 34/60), and older men had the lowest (18%, 19/104) (*p*<0.001). The median PrEP pill coverage for all participants was 40% (IQR 10%–70%), with the younger subgroups having lower pill coverage (median 10%, IQR 10%–70%) compared to the older subgroups (median 40%, IQR 10%–70%).

**Table 3:** PrEP continuation and adherence among pharmacy clients across the pilot duration

	All clients (*N*=287)	Clients <25 years		Clients ≥25 years	
		Women (*N*=64)	Men (*N*=60)	Women (*N*=59)	Men (*N*=104)
**PrEP continuation**					
Any PrEP continuation^a^	159 (53%)	27 (42%)	28 (47%)	35 (59%)	69 (66%)
Two follow‐up visits	103 (36%)	19 (30%)	14 (23%)	24 (41%)	46 (44%)
Three follow‐up visits^b^	51 (18%)	9 (14%)	9 (15%)	11 (19%)	22 (21%)
Stopped & restarted PrEP^c^	61 (21%)	11 (17%)	16 (27%)	15 (25%)	19 (18%)
**PrEP adherence**					
Overall pill coverage^d^					
Median % (IQR)	40% (10%−70%)	10% (10%−70%)	10% (10%−70%)	40% (10%−70%)	40% (10%−70%)
*Mean % (STD)*	*44% (35%)*	*37% (34%)*	*38% (34%)*	*46% (35%)*	*52% (35%)*

Abbreviations: IQR, interquartile range; PrEP, pre‐exposure prophylaxis; STD, standard deviation.

^a^ We defined “any PrEP continuation” as the % of participants who returned to the pharmacy and refilled PrEP at any point during follow‐up.

^b^ Percentages for three follow‐up visits were among the total number of participants eligible for the 7‐month visit (*n*=242).

^c^ We defined “stopped & restarted PrEP” as returning for a PrEP follow‐up visit 15 or more days after the scheduled visit and more than 15 days prior to the next scheduled visit and refilling PrEP medication. We calculated percentages of those who returned to at least one visit and were eligible to stop/restart.

^d^ We calculated overall pill coverage by dividing the person‐days of PrEP coverage by the person‐days of PrEP use. Person‐days of PrEP coverage = number of days of PrEP a participant has based on the total number of pills dispensed to them over the pilot duration. Person‐days of PrEP use = number of days a participant needed PrEP for effective coverage, which we equated to the total days of potential follow‐up for all enrolled participants (e.g. up to 300 days, which is 3 months following the 7‐month visit), with variations on when participants started PrEP and their potential follow‐up based on that date.

In multivariable analyses, being young (<25 years old) and inconsistently using condoms were significantly associated with a decreased likelihood of any PrEP continuation (Table 4). No factors were associated with stopping and restarting PrEP (Table S1). No pharmacy PrEP clients HIV seroconverted during the pilot duration.

**Table 4:** Correlates of any PrEP continuation among all pharmacy clients (*N*=287)

	Any continuation *n*/*N* (%)	Univariable^a^		Multivariable^a^	
		*PR (95% CI)*	*p‐value*	*aPR (95% CI)*	*p‐value*
Demographics					
Age <25 years	57/125 (46%)	0.72 (0.61–0.84)	0.000	0.78 (0.64–0.97)	0.022
Female	63/124 (51%)	0.85 (0.69–1.05)	0.141	0.89 (0.72–1.09)	0.268
Married	68/108 (63%)	1.22 (1.06–1.41)	0.005	1.02 (0.87–1.20)	0.766
Currently not in school	126/208 (61%)	1.41 (1.09–1.85)	0.014	1.26 (0.98–1.62)	0.070
Income >median, KES^b^	84/140 (60%)	1.16 (1.03–1.31)	0.014	0.96 (0.82–1.11)	0.544
Behaviours associated with HIV risk (past 6 months)					
Has a primary sexual partner	111/192 (58%)	1.12 (0.74–1.70)	0.590	–	–
Has a partner of unknown HIV status	47/90 (52%)	0.91 (0.69–1.21)	0.517	0.90 (0.71–1.14)	0.403
Has multiple sexual partners	60/101 (59%)	1.10 (0.99–1.24)	0.085	1.04 (0.98–1.12)	0.194
Has used EC ≥2 times	31/57 (54%)	0.97 (0.71–1.32)	0.846	–	–
Sells or buys sex (past 6 months)	44/81 (54%)	0.96 (0.79–1.17)	0.719	–	–
Has sex with alcohol (past 6 months)	54/101 (53%)	0.94 (0.71–1.24)	0.657	–	–
Inconsistently uses condoms	99/183 (54%)	0.92 (0.85–1.01)	0.068	0.89 (0.43–0.84)	0.003
Health seeking & PrEP knowledge					
Retail pharmacy is first place of healthcare seeking	86/155 (55%)	0.99 (0.76–1.28)	0.938	–	–
Travel time to pharmacy <15 minutes	65/121 (54%)	0.94 (0.69–1.27)	0.684	–	–
Came to the pharmacy seeking an SRH service^c^	138/242 (57%)	1.17 (0.88–1.54)	0.278	–	–
Learned of PrEP from word‐of‐mouth referral	52/101 (51%)	0.89 (0.62–1.27)	0.508	–	–
Knows someone taking PrEP	58/106 (55%)	0.97 (0.78–1.21)	0.792	–	–

Abbreviations: aPRs, adjusted prevalence ratios; EC, emergency contraception; KES, Kenyan shillings; PrEP, pre‐exposure prophylaxis; PRs, prevalence ratios; SRH, sexual and reproductive health.

^a^ Prevalence ratios estimated using GLM univariable and multivariable regression models with Poisson distribution and robust standard errors. We decided a prior that age, sex, marital status and having a partner of unknown HIV status were to be included in the multivariable analysis as well as associations with a *p*<0.10 in the univariable analyses.

^b^ Median monthly income across participants was 6245 KES.

^c^ SRH services sought included family planning, sexual performance enhancing drug, HIV self‐test, pregnancy test or PrEP.

### 3.3 Implementation outcomes

Clients’ perceived acceptability and appropriateness of pharmacy‐delivered PrEP services was very high across pharmacy PrEP visits; >95% of clients reporting that they agreed or strongly agreed with statements assessing different outcome component constructs (Table 5). Most clients (97%, 277/287 at initiation) indicated a willingness to pay for pharmacy‐delivered PrEP services. At initiation, the median amount clients reported being willing to pay was 300 KES (IQR 150–450 KES), equivalent to $2.71 USD (IQR $1.81–$4.52 USD); an amount that stayed consistent at 1 month, then decreased at subsequent follow‐up visits. For outcomes by age and sex, see Table S2.

**Table 5:** Implementation outcomes associated with pharmacy‐delivered PrEP services among clients over the pilot duration

		Initiation (*N*=287)	Month 1 (*N*=153)	Month 4 (*N*=103)	Month 7 (*N*=51)
Acceptability^a^					
Liked pharmacy‐delivered PrEP services	*Strongly agree or agree, n (%)*	283 (99%)	153 (100%)	103 (100%)	51 (100%)
Pharmacy‐delivered PrEP services is easy/not complicated	*Strongly agree or agree, n (%)*	277 (97%)	152 (99%)	103 (100%)	50 (98%)
Would like to continue pharmacy‐delivered PrEP services	*Strongly agree or agree, n (%)*	285 (99%)	153 (100%)	103 (100%)	51 (100%)
Would recommend pharmacy‐delivered PrEP services	*Strongly agree or agree, n (%)*	282 (98%)	153 (100%)	99 (96%)	51 (100%)
Appropriateness^b^					
Pharmacy‐delivered PrEP services fits my needs	*Strongly agree or agree, n (%)*	–	151 (99%)	101 (98%)	51 (100%)
Pharmacy‐delivered PrEP services is a good match for my needs	*Strongly agree or agree, n (%)*	–	151 (99%)	103 (100%)	51 (100%)
Costs					
Willingness to pay for pharmacy‐delivered PrEP services, *n* (%)		277 (97%)	150 (98%)	99 (96%)	48 (94%)
Amount willing to pay,^c^ median (IQR)	Kenyan shillings	300 (200–500)	300 (200–375)	200 (100–300)	150 (100–200)
	*US dollars* ^d^	*$2.71 ($1.81–4.52)*	*$2.71 (1.81–3.39)*	*$1.81 (0.90–2.71)*	*$1.35 (0.90–1.81)*

Abbreviations: IQR, interquartile range; PrEP, pre‐exposure prophylaxis.

^a^ Our assessment of acceptability was based on different component constructs defined within the Theoretical Framework of Acceptability (TFA): likes, intentions to continue and would recommend to a friend (TFA construct: affective attitude); easy/not complicated (TFA construct: burden component).

^b^ Our assessment of appropriateness was based on the Intervention Appropriateness Measure (IAM). Appropriateness was only measured at follow‐up visits.

^c^ We only report the amount willing to pay among those willing to pay a non‐zero value for pharmacy‐delivered PrEP services at each study visit.

^d^ We converted from Kenya shillings (KES) to US dollars (USD) using conversion rate averaged from 11/2020 to 12/2021 ($1USD = 110.72 KES); https://www.exchangerates.org.uk/KES‐USD‐spot‐exchange‐rates‐history‐2020.html.

## 4 DISCUSSION

The delivery of daily, oral PrEP for HIV prevention at private, community‐based pharmacies is new for Africa. This novel differentiated service delivery model reached individuals at HIV risk and a group distinct from those seeking PrEP services at public healthcare facilities. Notably, this model reached older men—a population that does not commonly engage with traditional, facility‐based PrEP services [40]. Additionally, PrEP initiation and continuation outcomes in this pilot were comparable to or higher than those generally observed at public healthcare facilities in Kenya [31]. Clients perceived pharmacy‐delivered PrEP services as highly acceptable and appropriate and were willing to pay for PrEP services in this setting.

When Kenya first rolled out PrEP nationally, HIV‐negative individuals in serodiscordant relationships were identified as those who could benefit most from PrEP services; thus, PrEP service delivery focused on integration within public HIV clinics. In many facility‐based PrEP implementation projects in Kenya, most PrEP clients have been women, individuals ≥25 years old and married people [31, 41, 42, 43, 44, 45]. By contrast, over half of the clients initiating PrEP in this pilot were men, almost half were <25 years old and less than half were married. Additionally, PrEP clients at pharmacies reported a higher prevalence of behaviours associated with HIV risk (e.g. recent sex with a partner of unknown HIV status and multiple sexual partners) than those at public healthcare facilities [40, 46].

In this pilot, roughly two‐thirds of men initiating PrEP were ≥25 years old and, compared to other subgroups, older men had the highest PrEP continuation. This suggests that pharmacy‐delivered PrEP services may address a previously unmet need among older men who are interested in and could benefit from PrEP. Reaching this population is especially important in Africa, where young women [47, 48]—a priority HIV prevention population [49, 50]—often acquire HIV from older male partners. Additionally, these men are where the HIV epidemic is likely to persist when population levels of HIV treatment and prevention coverage are high [51]. Pharmacy‐delivered PrEP services may also be a good fit for older men who, compared to other populations, are more likely to have disposable income that could be used to purchase PrEP services.

The levels of PrEP initiation and continuation at private pharmacies in this pilot were similar to or exceed those observed at public healthcare facilities in Kenya [8, 10, 15, 16, 17]. In the Partners Scale‐up Project, an implementation study of PrEP delivery at 25 public HIV clinics in Kenya over 30 months (January 2017–June 2019), PrEP continuation was 57% (2806/4898), 44% (2135/4898) and 34% (1661/4898) at 1, 3 and 6 months [40]—similar to what was observed in this pilot [40]. In other facility‐based PrEP implementation studies among women in Kenya over 7 months (November 2017–June 2018), PrEP initiation among those screened was 22% (278/1271) in family planning (FP) and 22% (2030/9376) in maternal and child health (MCH) clinics, and PrEP continuation was 41% (114/278) in FP and 39% (786/2030) in MCH clinics [41, 42]—similar to the subgroup of young women and lower than the subgroup of older women in this pilot. The achievement of similar levels of PrEP continuation in this pilot, even when clients were charged a fee for pharmacy‐delivered PrEP services, emphasizes the great interest in and need for PrEP services in this setting.

This study had limitations. Only five, carefully selected pharmacies in two counties participated in this pilot. Private pharmacies in Kenya vary widely in terms of size, clientele, geography and resources; more research is needed to understand how this model performs in different pharmacy settings. Age and sex were only collected among enrolees, thus preventing assessment of PrEP initiation by our subgroups of interest. Our model did not include STI testing. Despite the great need, STI testing is rarely available at public healthcare facilities in Kenya [52], with most facility‐based PrEP clients receiving only syndromic STI screening and management; future iterations of this model could consider incorporating STI testing. Because utilization and implementation outcomes were only measured among pharmacy clients (and not providers) who initiated and continued pharmacy‐delivered PrEP services, we do not know why clients never initiated or discontinued pharmacy PrEP services. Finally, the COVID‐19 pandemic may have impacted our continuation outcomes, as Kenya experienced various lockdown periods, and some pharmacy clients may have relocated away from the pharmacy where they initiated PrEP [53].

For pharmacy‐delivered PrEP services to be sustainable over time, cost structures need to be considered. In this pilot, the cost of implementing pharmacy‐delivered PrEP services was offset by a monthly stipend to the pilot pharmacies for research activities, donated commodities from the Kenya MOH and a fee clients paid for each PrEP visit. As for‐profit entities, private pharmacies do not typically provide services free of charge. Additionally, clients expect to purchase services at pharmacies and often do so even when these same services are available for free at public facilities. Although a fee for pharmacy‐delivered PrEP services may exclude some populations that could benefit, it may increase access to new populations that would otherwise have never engaged in facility‐delivered PrEP services. To improve PrEP continuation in future model iterations, a public‐sector payer or implementor could consider fully or partially subsidizing these services. Such subsidization could come in a range of forms, such as commodity donation or issuing vouchers to specific PrEP priority populations (e.g. young women) so they can get PrEP at pharmacies for free or a reduced price.

For this model of pharmacy‐delivered PrEP services to be scaled up in Kenya and similar settings, new frameworks and innovations that can support task‐shifting PrEP prescribing to pharmacy providers and delivering public goods in private settings are needed. One mechanism used in the United States is a collaborative practice agreement, which enables pharmacy providers to initiate, adjust and discontinue specified prescription drugs under the remote supervision of a clinician [54]. Alternatively, formalized training curriculums could be developed to certify interested pharmacy providers in PrEP delivery, like the Pharmacy Initiated Management of Antiretroviral Treatment course in South Africa [21, 55]. New public–private partnership frameworks should also be considered to support the delivery of donated public antiretroviral medications [56] in private settings. Implementing such frameworks would likely require adapting or developing novel electronic monitoring systems for facilitating supply chain management, generating drug dispensing reports, monitoring for fraud and reimbursing private pharmacies for their assistance in delivering public goods [57, 58].

## 5 CONCLUSIONS

Pharmacy‐based PrEP delivery is an exciting new differentiated service delivery model that was feasible and acceptable in this Kenya‐based pilot and reached populations not engaged in traditionally delivered health services. However, to inform the implementation of this model at scale, we need to evaluate its performance in a greater number of pharmacies, compare its effectiveness to pharmacy referral to facility‐delivered PrEP services (i.e. assess the potential value‐add, if any, of expanding PrEP prescribing to pharmacy providers’ scope of practice), develop cost and incentive structures that are acceptable to clients and motivating to pharmacies, and establish frameworks for delivering public goods in private settings.

To end the AIDS epidemic, both innovative prevention products and delivery mechanisms are needed to reach the populations in which HIV persists. As new PrEP modalities are introduced to Africa (e.g. the vaginal ring [59, 60] and injectable PrEP [61, 62]), a key priority should be ensuring that these products are available in the community where people need them. Pharmacies are one promising option worthy of further consideration.

## COMPETING INTERESTS

JMB is an employee of Gilead Sciences, outside of the present work; he has also received donations of study medication from Gilead Sciences and serves on advisory committees for Gilead Sciences, Merck and Janssen. KN has received research funding from the Merck Investigators Studies Program. For the remaining authors, none were declared.

## AUTHORS’ CONTRIBUTIONS

KFO, EB, KN and JMB contributed to the study conception and design of this pilot study. PM and VO led recruitment and study operations. MA, SG, APK and KFO analysed the data and KFO wrote the first draft of this manuscript. All authors edited the draft, provided insights and approved the final manuscript for publication.

## FUNDING

This work was supported by the National Institute of Mental Health (R34 MH120106; PI: K Ortblad). Dr. Ortblad received additional funding from the National Institute of Mental Health (K99 MH121166; PI: K Ortblad) and the National Institute of Allergy and Infectious Disease (P30 AI027757; PI: C Celum).

## Supporting information


**Table S1**: Correlates of stopping & restarting PrEP among pharmacy clients, *N* = 287
**Table S2**: Willingness to pay for pharmacy‐based PrEP delivery among pharmacy clients over the pilot study duration

## Data Availability

The data that support the findings of this study are available on request from the corresponding author. The data are not publicly available due to privacy or ethical restrictions.
