# Correction to Stand‐alone model for pharmacy delivery of oral HIV pre‐exposure prophylaxis in Kenya: a single‐arm, prospective pilot evaluation.

**DOI:** 10.1002/jia2.26169

**Published:** 2023-08-29

Ortblad KF et al. Journal of the International AIDS Society 2023 :e26131

In the article title, the word ‘pharmacy’ was missing and has been added to the online version of the article.

The incorrect title reads:

Stand‐alone model for **delivery** of oral HIV pre‐exposure prophylaxis in Kenya: a single‐arm, prospective pilot evaluation

The correct title should read:

Stand‐alone model for **pharmacy delivery** of oral HIV pre‐exposure prophylaxis in Kenya: a single‐arm, prospective pilot evaluation.

We apologize for this error.

