# Supplementary material for: Stand‐alone model for delivery of oral HIV pre‐exposure prophylaxis in Kenya: a single‐arm, prospective pilot evaluation
Source: J Int AIDS Soc. 2023 Jun 12;26(6):e26131. doi: 10.1002/jia2.26131 (PMC10258863; doi:10.1002/jia2.26131)
Supplement: Supplementary file 1 — Table S1: Correlates of stopping & restarting PrEP among pharmacy clients, N = 287 Table S2: Willingness to pay for pharmacy‐based PrEP delivery among pharmacy clients over the pilot study duration [file JIA2-26-e26131-s001.docx]

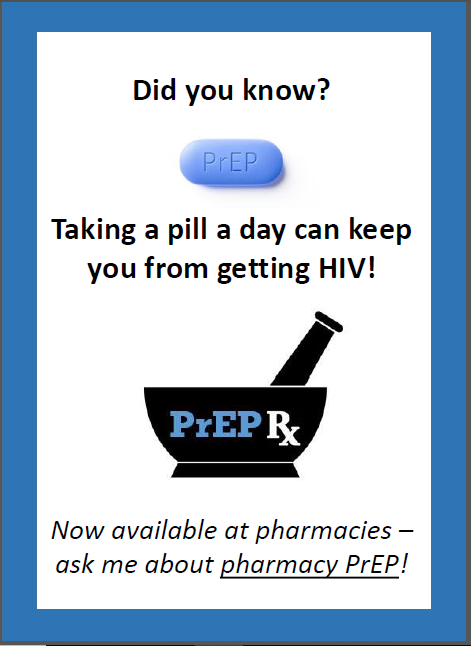


**Supporting Figure 1. Pharmacy PrEP poster advertisement displayed at pilot pharmacies**


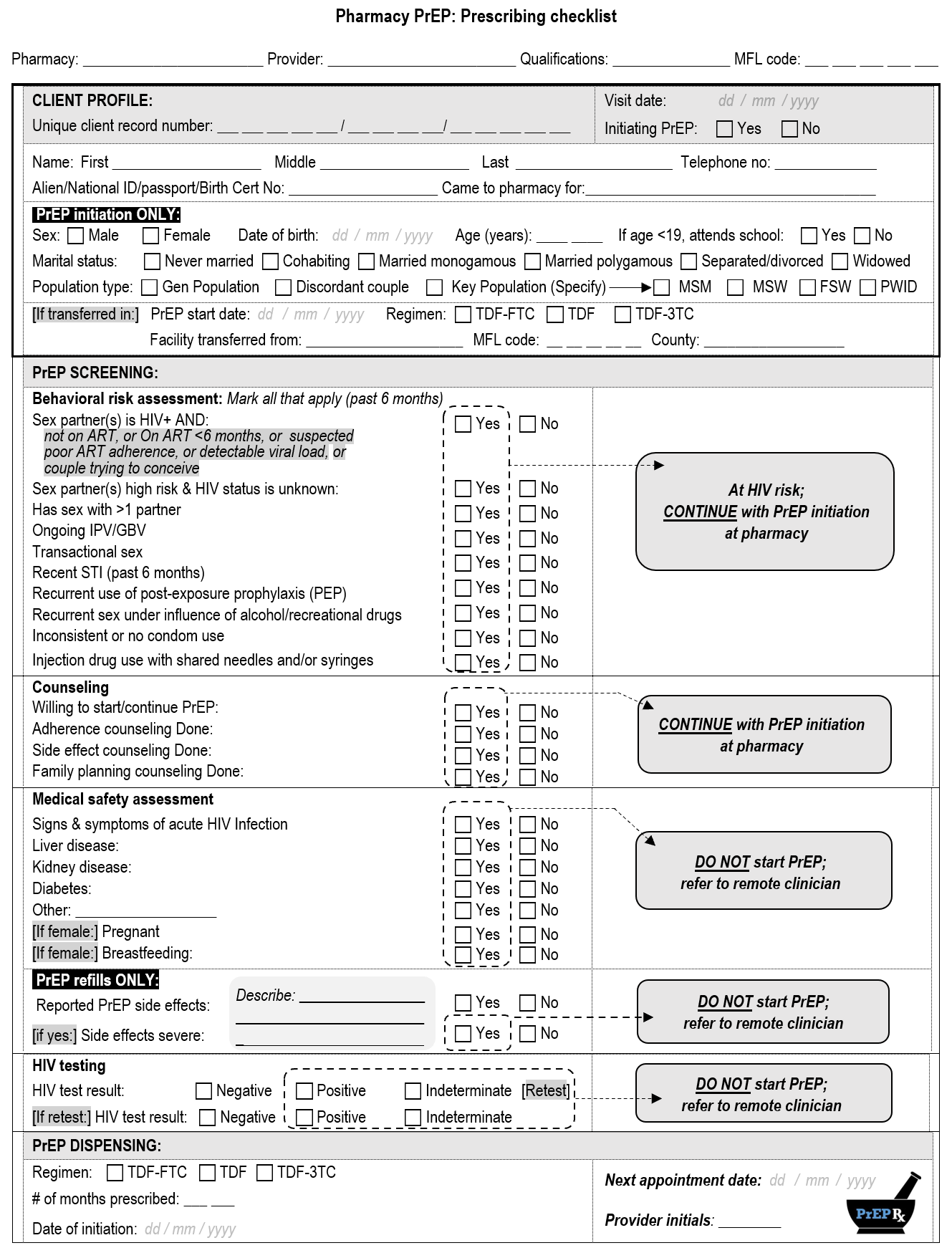


**Supporting Figure 2. Pharmacy-based PrEP delivery prescribing checklist**

| **Supporting Table 1. Correlates of stopping & restarting PrEP among pharmacy clients, N=287** | | | | | |
| --- | --- | --- | --- | --- | --- |
|  | **Stopping & restarting**  **n/N (%)** | **Univariable^1^** | | **Multiivariable^1^** | |
|  |  | *PR (95% CI)* | *p-value* | *aPR (95% CI)* | *p-value* |
| Clustered by pharmacy |  |  |  |  |  |
| Demographics  Age <25 years | 24/125 (19%) | 1.07 (0.70-1.64) | 0.746 | 0.95 (0.64-1.43) | 0.817 |
| Female | 23/124 (19%) | 1.01 (0.49-2.07) | 0.983 | 0.97 (0.46-2.08) | 0.948 |
| Married | 17/108 (16%) | 0.78 (0.49-1.25) | 0.305 | 0.76 (0.46-1.28) | 0.304 |
| Currently not in school | 35/208 (17%) | 0.74 (0.22-2.42) | 0.618 | - | - |
| Income >median, KES^2^ | 22/140 (16%) | 0.75 (0.37-1.52) | 0.417 | - | - |
| Behaviors Associated with risk (past 6 months) |  |  |  |  |  |
| Has a primary sexual partner | 37/192 (19%) | 1.14 (0.73-1.80) | 0.562 | - | - |
| Has a partner of unknown HIV status^2^ | 16/90 (18%) | 0.95 (0.66-1.36) | 0.767 | 0.21 (0.14-0.32) | 0.828 |
| Has multiple sexual partners | 19/101 (19%) | 1.03 (0.59-1.79) | 0.919 | - | - |
| Has used EC ≥2 times | 12/57 (21%) | 1.18 (0.92-1.51) | 0.186 | - | - |
| Sells or buys sex (past 6 months) | 14/81 (17%) | 0.91 (0.60-1.39) | 0.671 | - | - |
| Has sex with alcohol (past 6 months) | 18/101 (18%) | 0.95 (0.37-2.39) | 0.909 | - | - |
| Inconsistently uses condoms | 33/183 (18%) | 0.94 (0.51-1.71) | 0.835 | - | - |
| Health seeking & PrEP knowledge |  |  |  |  |  |
| Retail pharmacy is first place of healthcare seeking | 32/155 (21%) | 1.30 (0.77-2.18) | 0.326 | - | - |
| Travel time to pharmacy <15 mins | 23/121 (19%) | 1.05 (0.82-1.35) | 0.695 | - | - |
| Came to the pharmacy seeking an SRH service^3^ | 44/242 (18%) | 0.91 (0.52-1.60) | 0.740 | - | - |
| Learned of PrEP from word-of-mouth referral | 17/101 (17%) | 0.87 (0.56-1.34) | 0.526 | - | - |
| Knows someone taking PrEP | 23/106 (22%) | 1.31 (0.88-1.95) | 0.183 | - | - |
| **Abbreviations:** adjusted prevalence ratios (aPRs); emergency contraception (EC); Kenyan Shilling (KES); prevalence ratios (PRs); pre-exposure prophylaxis (PrEP); sexual and reproductive health (SRH).  ^1^Prevalence ratios estimated using GLM univariable and multivariable regression models with Poisson distribution and robust standard errors. We decided a prior that age, sex, marital status, and having a partner of unknown HIV status were to be included in the multivariable analysis as well as associations with a p<0.10 in the univariable analyses.  ^2^Median monthly income across participants was 6,245 KES.  ^3^SRH services sought included family planning, sexual performance enhancing drug, HIV self-test, pregnancy test, or PrEP. | | | | | |

| **Supporting Table 2. Willingness to pay for pharmacy-based PrEP delivery among pharmacy clients over the pilot study duration** | | | | |
| --- | --- | --- | --- | --- |
|  | **Initiation** | **Month 1** | **Month 4** | **Month 7** |
| **Clients <25 years:** |  |  |  |  |
| **Women** | **N=64** | **N=27** | **N=19** | **N=9** |
| Willingness to pay, n (%) | 62 (97%) | 24 (89%) | 16 (84%) | 8 (89%) |
| Amount willing to pay^1^ (KES), median (IQR) | 300 (150-400) | 300 (150-350) | 250 (100-400) | 300 (75-400) |
| **Men** | **N=60** | **N=27** | **N=14** | **N=9** |
| Willingness to pay, n (%) | 57 (95%) | 24 (89%) | 11 (79%) | 8 (89%) |
| Amount willing to pay^1^ (KES), median (IQR) | 300 (150-375) | 300 (200-450) | 250 (100-425) | 300 (100-450) |
| **Clients ≥25 years:** |  |  |  |  |
| **Women** | **N=59** | **N=32** | **N=24** | **N=11** |
| Willingness to pay, n (%) | 58 (98%) | 28 (88%) | 22 (92%) | 10 (91%) |
| Amount willing to pay^1^ (KES), median (IQR) | 300 (175-375) | 200 (200-350) | 300 (200-500) | 200 (200-350) |
| **Men** | **N=104** | **N=67** | **N=46** | **N=22** |
| Willingness to pay, n (%) | 100 (96%) | 55 (82%) | 43 (93%) | 21 (95%) |
| Amount willing to pay^1^ (KES), median (IQR) | 275 (150-500) | 250 (150-500) | 300 (200-500) | 300 (150-500) |
| **Abbreviations:** interquartile range (IQR); Kenyan shilling (KES).  ^1^Amount willing to pay measured among clients willing to pay a non-zero value for pharmacy-delivered PrEP services at each PrEP visit. | | | | |
|  | | | | |
